# Definition of trAnscatheter heart Valve orIeNtation in biCuspId aortic valve: The DA VINCI pilot study

**DOI:** 10.3389/fcvm.2022.1056496

**Published:** 2022-12-12

**Authors:** Giuseppe Tarantini, Tommaso Fabris, Luca Nai Fovino, Francesco Cardaioli, Valeria Pergola, Carolina Montonati, Giulio Rodinò, Giulio Cabrelle, Mauro Massussi, Andrea Scotti, Vittorio Zuccarelli, Tommaso Sciarretta, Giulia Masiero, Dario Gregori, Massimo Napodano, Chiara Fraccaro, Saverio Continisio, Sabino Iliceto

**Affiliations:** ^1^Department of Cardiac, Thoracic, Vascular Sciences and Public Health, University of Padua, Padua, Italy; ^2^Department of Medicine—DIMED, University of Padua, Padua, Italy; ^3^Cardiac Catheterization Laboratory and Cardiology, ASST Spedali Civili di Brescia, Department of Medical and Surgical Specialties, Radiological Sciences, and Public Health, University of Brescia, Brescia, Italy; ^4^Montefiore-Einstein Center for Heart and Vascular Care, Montefiore Medical Center, Albert Einstein College of Medicine, Bronx, NY, United States; ^5^Unit of Biostatistics, Epidemiology and Public Health, University of Padua, Padua, Italy

**Keywords:** transcatheter aortic valve replacement, bicuspid aortic valve, commissural alignment, coronary access, computed tomography

## Abstract

**Objectives:**

To assess the impact of conventional transcatheter heart valve (THV) commissural alignment techniques on THV/coronary overlap and coronary access (CA) after transcatheter aortic valve replacement (TAVR) in bicuspid aortic valve (BAV).

**Background:**

Specific Evolut Pro/Pro + and Acurate Neo2 THV orientations are associated with reduced neo-commissural overlap with coronary ostia in tricuspid aortic anatomy. Whether standard orientation techniques are effective also in the setting of BAV anatomy has not been studied.

**Methods:**

The DA VINCI (Definition of trAnscatheter aortic Valve orIeNtation in biCuspId aortic valve) pilot study is a prospective registry enrolling consecutive patients with severe BAV stenosis undergoing TAVR with last generation supra-annular tall-frame THVs implanted with a cusp overlap view-based commissural alignment. Patients underwent pre- and post-TAVR computed tomography (CT) and coronary angiography. The study endpoint was the rate of favorable THV/coronary overlap, defined as an angle > 40° between the THV commissural post and coronary ostia. Other endpoints were the rates of successful THV alignment with respect to the raphe and of selective CA after TAVR. Moreover, different virtual THV alignment models were tested to identify which one would produce the lower degree of THV/coronary overlap.

**Results:**

Thirty-four patients with type 1 BAV with right-left raphe undergoing TAVR (23 with Evolut Pro/Pro + and 11 with Acurate Neo2) were included. At pre-TAVR CT, moderate/severe cusp asymmetry was found in 50% of patients, severe coronary ostia eccentricity was observed in 47.1% for the RCA vs. 8.8% for the LCA (*P* < 0.007). Correct TVH orientation was achieved in 29 cases. At post-TAVR CT, optimal THV alignment/mild misalignment to the raphe was observed in 86.2%, but a moderate/severe overlap with the coronaries was seen in 13.7% for the RCA and 44.8% for the LCA (*P* = 0.019). After TAVR, selective RCA cannulation was possible in 82.8% vs. 75.9% for the LCA (*P* = 0.74), while combined selective CA of both coronaries was possible in less than two-thirds of the patients. Virtual THV alignment in the coronary ostia overlap view assuming a hypothetical circular THV expansion would produce an optimal THV/coronary overlap in almost 90% of cases.

**Conclusion:**

Given cusp asymmetry and coronary ostia eccentricity of BAV combined with potential THV asymmetrical expansion, conventional commissural alignment techniques are associated with higher rates of THV misalignment and of moderate/severe neo-commissure overlap with the coronary ostia as compared to tricuspid aortic stenosis, resulting in lower rates of selective CA after TAVR. A modified THV orientation technique based on the coronary ostia overlap view might be preferable in BAV patients.

## Introduction

Transcatheter aortic valve replacement (TAVR) is approved for low-risk and younger patients with severe aortic stenosis (AS). Given the longer life-expectancy of this population, the preservation of free coronary access (CA) for future coronary interventions is of utmost importance (1–3). Differently from surgical aortic valve replacement (SAVR), during TAVR the native aortic leaflets are not removed and there is the risk to randomly end up with a neo-commissure in front of coronary ostia and to interfere with coronary flow or cannulation (4, 5). Specific techniques to align the neo-commissures have been recently reported for supra-annular THVs, such as the Evolut R/Pro/Pro + (Medtronic) and the Acurate Neo/Neo2 (Boston Scientific) (6, 7), but these have been explored mostly in the setting of tricuspid aortic valve (TAV) (8, 9).

Less is known about the rate of successful commissural alignment with current implantation techniques in patients with bicuspid aortic valve (BAV) treated by TAVR. In fact, the anatomic variability of different BAV types, location of raphe and coronary ostia may increase the risk of THV misalignment with respect to the coronaries (10, 11). This concept has been recently described by Wang et al. in a pre-procedural computed tomography (CT) study, which showed that BAV carries higher cusp asymmetry and more pronounced coronary ostia eccentricity as compared to TAV (12). However, whether the anatomic variability of BAV represents a potential Achille’s heel of current commissural alignment techniques remains yet to be investigated. Thus, we aimed to prospectively evaluate the impact of a standard (i.e., for TAV) commissural alignment technique in the setting of type 1 BAV. Furthermore, we assessed the impact of BAV-related asymmetry on THV/coronary overlap and CA after TAVR by CT evaluation and selective coronary cannulation.

## Materials and methods

### Study population

The DA VINCI (Definition of trAnscatheter aortic Valve orIeNtation in biCuspId aortic valve) study is a single-center registry enrolling consecutive patients with severe bicuspid AS who underwent trans-femoral TAVR with last generation supra-annular tall-frame THVs implanted with commissural alignment. Only patients with type 1 BAV with right-left (R-L) raphe were finally considered for the study’s analyses (13). Patients without device success defined according to VARC-3 (Valve Academic Research Consortium) criteria were excluded (14). Indications for TAVR, approach, THV choice, and pre-TAVR percutaneous revascularization of severe coronary lesions were based on the local Heart Team’s judgement. Decision to pre-dilate the native aortic valve was left to operator’s preference. Post-dilation of the THV was performed in case of more than mild aortic regurgitation or significant residual gradient. Baseline clinical characteristics and procedural data were prospectively collected in a dedicated database. According to standard practice, all patients underwent pre-TAVR coronary angiography and CT assessment, while those with successful THV orientation based on fluoroscopic evaluation underwent also post-TAVR coronary angiography and CT (as per study’s purposes). All participants provided their informed consent for post-TAVR coronary angiography and CT, as well as for data collection. The study was conducted according to the principles of the Declaration of Helsinki and Good Clinical Practice.

### Transcatheter heart valve design

Two types of THV were included in this study. The supra-annular self-expanding Evolut Pro/Pro + (Medtronic, Ireland) has a tall frame with a small diamond cell design extending above the coronaries and a commissural post height of 26 mm. The supra-annular self-expanding Acurate Neo2 valve (Boston Scientific, USA) has a tall frame with an open cell architecture in its upper part and a commissural post height of 28-31 mm, according to valve size.

### Commissural alignment

**FIGURE 1:**
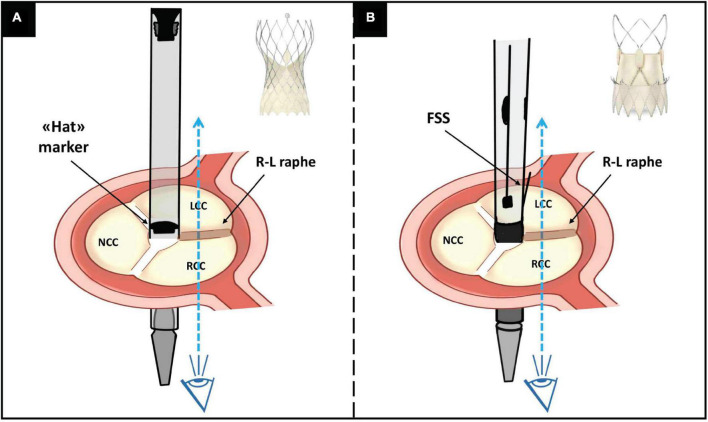
Illustration of the R-L cusp overlap orientation technique for: **(panel A)** Evolut and **(panel B)** Acurate Neo2 THV. See the description in the main text. R, right; L, left; THV, transcatheter heart valve; NCC, non-coronary cusp; RCC, right coronary cusp; LCC, left coronary cusp; FSS, free stent strut.

All implants were performed attempting commissural alignment based on previously described techniques for TAV (Figure 1) (6, 7):

(a). The Evolut Pro/Pro + system was inserted with the flush port of the delivery catheter positioned at 3 o’clock (i.e., away from the operator) and the position of the “Hat” marker was firstly checked in the descending aorta. If the “Hat” marker was not located at the outer curve (OC) of the aorta, the delivery system was rotated counterclockwise (or clockwise depending on vessel tortuosity) to achieve the desired orientation before advancing the valve into final position. Then, orientation of the “Hat” marker was checked in the R-L cusp overlap view (usually a right anterior oblique and caudal projection), which displays the R-L raphe on the right side of the screen, aiming for the “Hat” marker to be in center front (CF) position. If the “Hat” marker was located at the inner curve or center back, the valve was retrieved in the descending aorta and an attempt to rotate the delivery system more counterclockwise (or clockwise depending on vessel tortuosity) was made. Fluoroscopic commissural orientation for Evolut Pro/Pro + was defined favorable if the “Hat” marker was positioned at the CF in the R-L cusp overlap view.

(b). The Acurate Neo2 system was inserted with the flush port in the 6 o’clock position. The prosthesis was then advanced in the final position and, before crossing the aortic valve, orientated to achieve one free stent strut (FSS) (corresponding to one of the commissural posts) facing the IC of the aorta (right side of the screen) in the R-L cusp overlap view.

### Coronary access after transcatheter aortic valve replacement

Selective cannulation of right (RCA) and left coronary artery (LCA) from the trans-femoral access was always attempted before and after TAVR. All angiographic images were independently reviewed by two experienced interventional cardiologists (L.N.F. and G.T.). A first attempt to cannulate the coronaries was made with standard diagnostic catheters (Judkins Right and Left – Cordis, USA). If unsuccessful, a different diagnostic or guiding catheter was used, according to operators’ preference. No further cannulation attempt with the use of a coronary guidewire nor guide-extension catheter was performed in case of unfeasible re-access to coronary, as per study protocol. Coronary access was defined selective if successful intubation of the coronary ostium was achieved, sub-selective if the coronary artery could be displayed and adequately evaluated although without complete engagement of the coronary ostium, unfeasible if the coronary artery could not be adequately displayed (15).

### Pre- and post-transcatheter aortic valve replacement computed tomography evaluation

**FIGURE 2:**
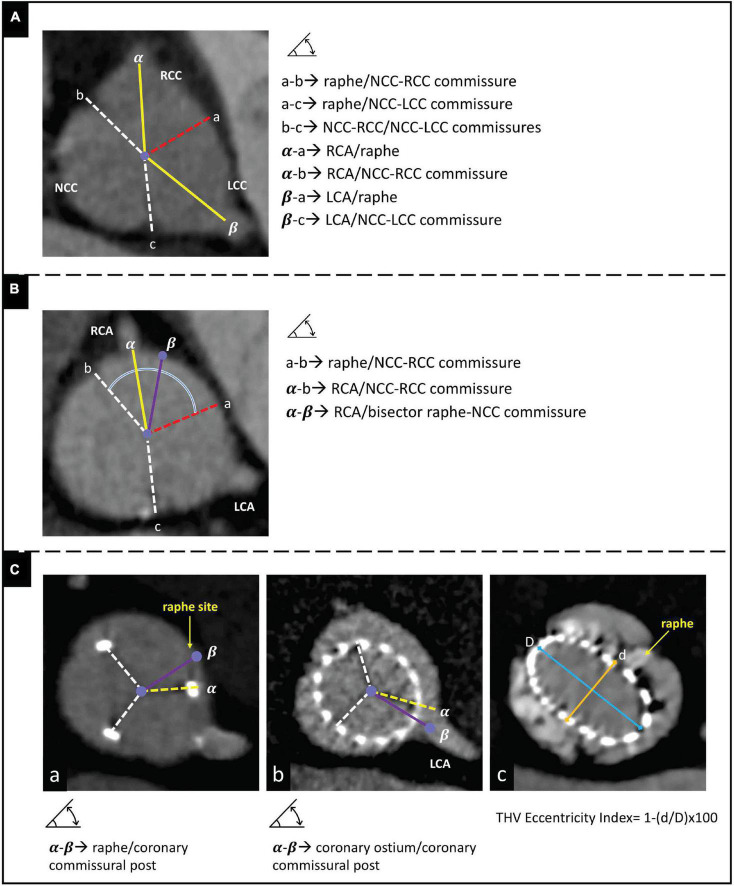
Panels **(A,B)** illustrate the measures assessed at pre-procedural CT scan to evaluate cusp symmetry and coronary ostia eccentricity, respectively. Panel **(C)** illustrates the measures assessed at post-procedural CT scan to evaluate: **(box a)** THV alignment, **(box b)** THV/coronary overlap, and **(box c)** THV circularity. CT, computed tomography; NCC, non-coronary cusp; RCC, right coronary cusp; LCC, left coronary cusp; RCA, right coronary cusp; LCA, left coronary cusp; THV, transcatheter heart valve; D, maximal diameter; d, minimal diameter.

Pre-TAVR CT measurements were conducted as described elsewhere (16). Standard series of measured parameters included coronary ostia height and ascending aortic diameter. The morphology of BAV was classified using the Sievers and Schmidtke (13) and TAVR-specific classification by Jilaihawi et al. (17). Additional measures, which were evaluated in the 50-70% phase of the cardiac cycle at the supra-annular level, included: the angle between the non-fused commissures, the angle between the non-fused commissures and the raphe, the angle between the coronary ostia and the non-fused commissures, and the angle between the coronary ostia and the raphe (Figure 2A). As previously described (12), based on the angle between the commissures delimiting the largest cusp (non-coronary cusp – NCC, or right coronary cusp – RCC, or left coronary cusp – LCC), BAVs were classified as: a) symmetric (120°-125°); b) mildly asymmetric (125°-135°); c) moderately asymmetric (130°-135°); and d) severely asymmetric (> 135°). Likewise, based on the angle deviation between each coronary ostium and the bisector of the corresponding cusp, the eccentricity of coronary ostia was classified as (12): a) centered (0°-10°); b) mildly eccentric (10-20°); moderately eccentric (20°-30°); and d) severely eccentric (>30°) (Figure 2B).

At post-TAVR CT, the observed final position of THV’s neo-commissures in relation to coronary ostia and raphe was determined (Figure 2C; boxes a-b). As previously described (6), the overlap between coronary ostium and neo-commissures (THV/coronary overlap) was considered severe when < 20°, moderate when 20-40°, and optimal when > 40°. THV alignment with respect to the prespecified target (i.e., raphe) was arbitrarily defined as: (a) optimal alignment (angle deviation 0-15°); (b) mild misalignment (angle deviation 15°-30°); (c) moderate misalignment (angle deviation 30°-45°; and (d) severe misalignment (angle deviation > 45°) (18). Supplementary measures included the distance between the THV frame and both coronary ostia, and the prosthesis’s eccentricity index (calculated as [THV minimum diameter/THV maximum diameter – 1] × 100 at the level of maximal raphe’s protrusion) (Figure 2C; box c). A THV was considered circular with an eccentricity index < 10% (19).

In addition, in a pre-procedural CT-based virtual THV modeling, the degrees of overlapping between coronary ostia and commissural posts were also assessed assuming four different scenarios of THV alignment: 1) THV orientation using the RCA/LCA coronary ostia overlap view with perfect alignment with respect to the bisector between the coronaries and a circular shaping to guarantee equal angle (120°) between the commissural posts; 2) THV orientation using the RCA/LCA coronary ostia overlap view with a perfect alignment with respect to the bisector between the coronaries, but applying the angles between the neo-commissures as observed at post-TAVR CT scan (thus accounting for possible THV underexpansion); 3) THV orientation using the standard R-L cusp overlap view with a perfect alignment with respect to the raphe (e.g. using the standard cusp overlap view) and a circular shaping to guarantee equal angle (120°) between the commissural posts; 4) THV orientation using the standard R-L cusp overlap view with a perfect alignment as respect to the raphe, but applying the angles between the neo-commissures as observed at post-TAVR CT scan (thus accounting for possible THV underexpansion). All post-TAVR CT scans were evaluated in the 50-70% phase of the cardiac cycle to better visualize the THV neo-commissures.

Pre- and post-TAVR CT scans were independently evaluated by two experienced cardiac CT reviewers (T.F. and L.N.F.) who were unaware of patient clinical data, while final agreement with a third expert reviewer (G.T.) was achieved in case of discordancy. All the analyses were performed using Aycan OsiriX Pro workstation (Aycan Medical Systems, Rochester, NY, USA).

### Study endpoints

The study endpoint was the rate of favorable THV orientation, defined as an angle > 40° between the THV commissural post and coronary ostia at post-TAVR CT. Other endpoints were the rate of successful THV alignment, that is the closest commissural tab to the raphe being positioned within ± 15°, and the rate of successful CA after TAVR. Moreover, the four different CT-based pre-specified THV alignment models were tested to identify which one would produce the most favorable THV/coronary overlap.

### Statistical analysis

Baseline characteristics are described with mean ± standard deviation (SD) or medians and 1st and 3rd interquartile ranges (IQRs) for continuous variables and percentages for discrete variables. Differences were detected using Student’s t-test for continuous variables and the chi-square or Fisher exact test for categorical variables. For all the analyses, a two-sided p < 0.05 was significant. Statistical analyses were performed using R, version 4.0.2 (R Foundation).

## Results

### Baseline characteristics

Thirty-four patients with severe AS and type 1 BAV undergoing TAVR were included in the study. Baseline clinical characteristics of the population are described in Table 1. Mean age was 79.3 ± 5.2 years. Mean Society of Thoracic Surgeons Predicted Risk of Mortality score was 5.7 ± 4.2%. Severe coronary artery disease was present in 14 (41.2%) patients, while 8 (23.5%) underwent a staged percutaneous coronary revascularization before TAVR.

**TABLE 1:** Study population characteristics.

	Overall population (*n* = 34)
**Clinical characteristics**	
Age, y	79.3 ± 5.2
Male	21 (61.8)
Dyslipidaemia	23 (67.6)
Hypertension	31 (91.2)
Diabetes mellitus	6 (17.6)
eGFR < 60 ml/min/1.73 mq	13 (38.2)
Previous TIA/stroke	2 (5.9)
Peripheral vascular disease	6 (17.6)
Previous MI	8 (23.5)
Previous cardiac surgery	5 (14.7)
Previous CABG	4 (11.8)
Prior PM	0 (0)
STS-PROM score	5.7 ± 4.2
**Coronary angiography**	
Severe CAD	14 (41.2)
Staged PCI pre-TAVR	8 (23.5)
**Echocardiography**	
LV-EF,%	58 (19-73)
Maximal aortic gradient, mmHg	74 (28-151)
Mean aortic gradient, mmHg	43 (14-91)
Mean ascending aorta diameter, mm	38 (22-49)

Values are mean ± SD, *n* (%), or median (range). eGFR, estimated glomerular fractional rate; TIA, transient ischemic attack; MI, myocardial infarction; CABG, coronary artery bypass graft surgery; PM, pacemaker; STS-PROM score, Society of Thoracic Surgeons Predicted Risk of Mortality score; CAD, coronary artery disease; PCI, percutaneous coronary intervention; TAVR, transcatheter aortic valve replacement; LV-EF, left ventricle ejection fraction.

### Pre-TAVR computed tomography

**FIGURE 3:**
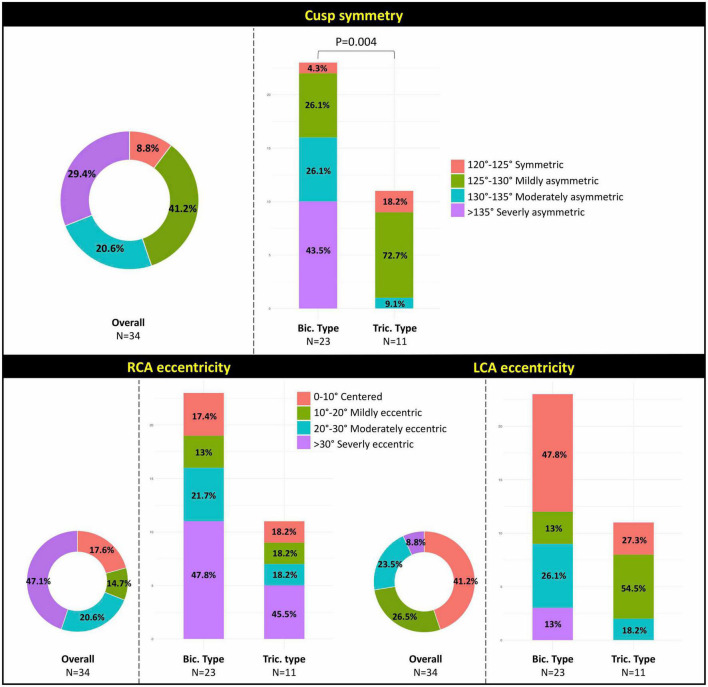
Illustration of the degree of anatomical asymmetry in study’s BAV population: **(top panel)** cusp symmetry; **(bottom panel)** coronary ostia eccentricity. BAV, bicuspid aortic valve; RCA, right coronary artery; LCA, left coronary artery; Bic., bicommissural; Tric., tricommissural.

Morphological characteristics of BAVs are presented in Table 2. All BAVs included in the study were type 1 of Siviers and Schmidtke classification (13) with R-L fusion, of which 23 (67.6%) were bicommissural type and 11 (32.4%) were tricommissural type with fibro-calcific fusion (17). Almost 50% of BAVs were moderately or severely asymmetric, with more pronounced asymmetry in the bicommissural vs. tricommissural type (*P* = 0.004) (Figure 3, top panel). Median coronary ostium height was 15.7 mm [10.1, 19.2] for the RCA and 18.0 mm [9.9, 25.7] for the LCA, without significant differences among BAV types. Overall, a severe coronary ostium eccentricity was more frequently encountered for the RCA than LCA (67.6 vs. 32.4%; *P* = 0.007) (Figure 3, bottom panel).

**TABLE 2:** Pre-procedural CT analysis – Overall study population.

	Overall (*n* = 34)	CoreValve Evolut (*n* = 23)	Acurate Neo2 (*n* = 11)	*P* value	Bicommissural type (*n* = 23)	Tricommissural type (*n* = 11)	*P* value
BAV type (TAVR classification)					−	−	−
Bicommissural	23 (67.6)	17 (73.9)	6 (54.5)	0.43			
Tricommissural	11 (32.4)	6 (26.1)	5 (45.5)				
BAV sub-type				−			−
R-L raphe	34 (100)	23 (100)	11 (100)		23 (100)	11 (100)	
R-N raphe	0 (0)	0 (0)	0 (0)		0 (0)	0 (0)	
L-N raphe	0 (0)	0 (0)	0 (0)		0 (0)	0 (0)	
Cusp symmetry							
Symmetric	3 (8.8)	1 (4.3)	2 (18.2)		1 (4.3)	2 (18.2)	
Mildly asymmetric	14 (41.2)	10 (43.5)	4 (36.4)	0.67	6 (26.1)	8 (72.7)	**0.004**
Moderately asymmetric	7 (20.6)	5 (21.7)	2 (18.2)		6 (26.1)	1 (9.1)	
Severely asymmetric	10 (29.4)	7 (30.4)	3 (27.3)		10 (43.5)	0 (0)	
RCA height, mm	15.7 (10.1-19.2)	15.4 (10.1-19.2)	16.1 (10.6-18.5)	0.74	16.1 (10.6-18.5)	15.4 (10.1-19.2)	0.54
LCA height, mm	18.0 (9.9-24.7)	17.9 (9.9-24.7)	18.1 (12.2-21.1)	0.69	19.5 (14.7-24.7)	16.6 (9.9-24.0)	0.06
RCA eccentricity							1.0
Centered	6 (17.6)	5 (21.7)	1 (9.1)		4 (17.4)	2 (18.2)	
Mild	5 (14.7)	3 (13)	2 (18.2)	0.87	3 (13)	2 (18.2)	
Moderate	7 (20.6)	5 (21.7)	2 (18.2)		5 (21.7)	2 (18.2)	
Severe	16 (47.1)	10 (43.5)	6 (54.5)		11 (47.8)	5 (45.5)	
LCA eccentricity							
Centered	14 (41.2)	10 (43.5)	4 (36.4)	0.86	11 (47.8)	3 (27.3)	0.10
Mild	9 (26.5)	5 (21.7)	4 (36.4)		3 (13)	6 (54.5)	
Moderate	8 (23.5)	6 (26.1)	2 (18.2)		6 (26.1)	2 (18.2)	
Severe	3 (8.8)	2 (8.7)	1 (9.1)		3 (13)	0 (0)	
Angle RCA/raphe,°	74.7 (35.6-119.5)	72.8 (35.6-119.5)	81.6 (62.1-107.3)	0.11	75.5 (35.6-119.5)	74.7 (62.1-85.4)	0.57
Angle RCA/NCC-RCC,°	47.2 (11.8-65.1)	47.6 (11.8-64.2)	46.2 (35.5-65.1)	0.50	46.2 (11.8-65.1)	50.9 (36.7-58.9)	0.45
Angle LCA/raphe,°	59.1 (40.2-103.4)	60.1 (40.4-103.4)	58.9 (40.2-81.0)	0.54	58.9 (40.4-103.4)	63.5 (40.2-69.7)	0.78
Angle LCA/NCC-LCC,°	52.0 (38.5-75.0)	50.5 (38.5-69.0)	55.7 (38.5-74.0)	0.37	49.8 (38.5-68.3)	55.7 (50.2-75.0)	**0.01**
Angle raphe/NCC-RCC,°	123.3 (93.9-151.4)	124.0 (93.9-132.2)	122.1 (112.8-151.4)	0.28	122.6 (93.9-151.4)	1255 (112.8-130.2)	0.98
Angle raphe/NCC-LCC,°	114.3 (96.9-149.9)	113.3 (97.3-149.9)	115.7 (96.9-127.8)	0.62	110.4 (96.9-149.9)	117.0 (109.9-127.8)	0.12
Angle NCC-RCC/NCC-LCC,°	121.3 (91.9-166.3)	123.1 (91.9-166.3)	119.4 (106.2-142.1)	0.15	123.1 (91.9-166.3)	120.4 (112.8-124.6)	0.35
Angle RCA/LCA,°	136.3 (85.6-184.8)	135.9 (85.6-184.8)	140.9 (123.4-157.4)	0.43	138.0 (85.6-184.8)	132.7 (111.7-145.5)	0.31
Bisector RCA/LCA,°	68.2 (42.8-92.4)	68.0 (42.8-92.4)	70.4 (61.7-78.7)	0.43	69.0 (42.8-924)	66.4 (55.9-72.7)	0.31
Angle deviation raphe/bisector RCA-LCA,°	8.4 (0.2-32.3)	7.2 (0.2-27.1)	13.0 (0.6-32.3)	0.50	9.1 (0.2-32.3)	7.6 (0.6-22.6)	0.37

Values are n (%), or median (range). CT, computed tomography; BAV, bicuspid aortic valve; R, right; L, left; N, non-coronary; RCA, right coronary artery; LCA, left coronary artery; NCC, non-coronary cusp; RCC, right coronary cusp; LCC, left coronary cusp. Bold values are those statistically significant.

The coronary ostia-to-raphe angle was wider than the coronary ostia-to-non-fused commissure angle (median angle 74.7° vs. 47.2° for the RCA and 59.1° vs. 52.0° for the LCA, respectively). The angle between the LCA ostium and the closest non-fused commissure was significantly wider in the bicommissural vs. tricommissural type (*P* = 0.01). Median angle between the coronary ostia was 136.3° [85.6, 184.8], with the bisector >60° in 29 cases (85.3%), >70° in 26 cases (76.5%), and >80° in 3 cases (8.8%). Median angle deviation between the raphe and the RCA/LCA bisector was 8.4° [0.2, 32.3]. Supplementary Figure 1 depicts the distribution of the variables measured at basal CT scan according to BAV type.

### Transcatheter aortic valve replacement procedure

Table 3 reports the data relative to TAVR procedure and THV orientation. Transfemoral access was used in 100% of procedures. The Evolut Pro/Pro + was implanted in 23 patients, the Acurate Neo2 in the remaining 11 subjects. As per inclusion criteria, procedural success was achieved in all cases. Overall, pre- and post-dilatation were performed in 34 (100%) and 12 (35%) of cases, respectively. A supra-annular approach for THV sizing was applied in 20 (59%) implants. Successful fluoroscopic orientation was achieved in 29 cases (85.3%), of which 19 (82.6%) with the Evolut Pro/Pro + and 10 (90.9%) with the Acurate Neo2 (*P* = 1.0). Reported reasons for unsuccessful THV orientation were the presence of severe tortuosity of ilio-femoral axis in 3 of the 5 cases (all using the CoreValve Evolut sheath-less delivery) and the presence of severe aortic angulation (>70°) in 2 cases (one per prosthesis type), which hindered the delivery catheter’s free rotation.

### Coronary angiography after transcatheter aortic valve replacement

**FIGURE 4:**
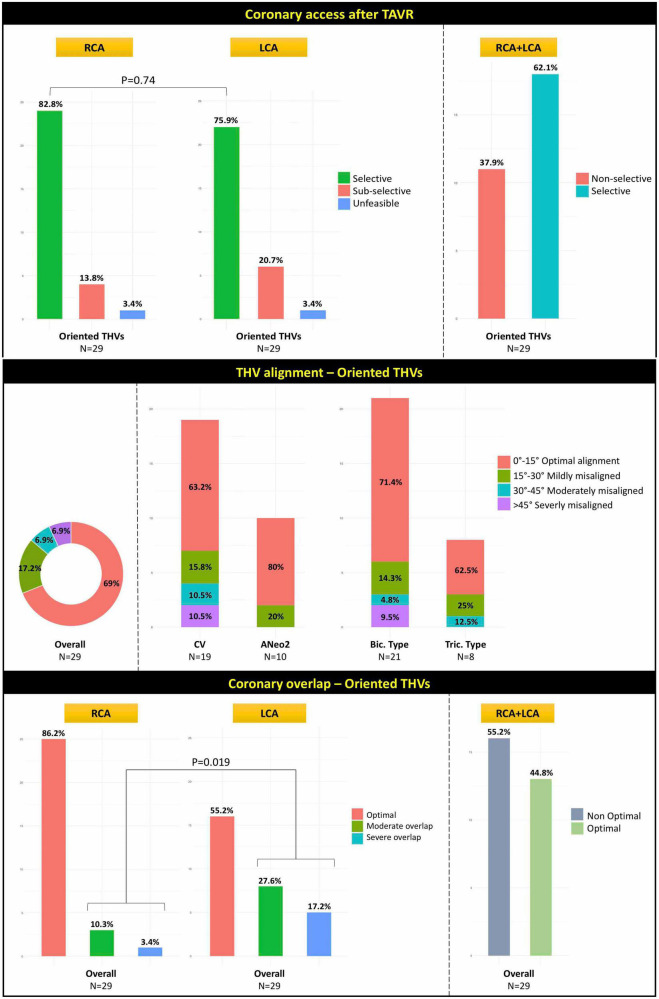
Illustration of the rates of CA after TAVR for RCA, LCA, and combined RCA/LCA **(top panel)**, rates of the degree of THV alignment **(middle panel)**, and neo-commissural/coronary overlap **(bottom panel)** in the 29 patients with fluoroscopically oriented THV. CA, coronary access; TAVR, transcatheter aortic valve replacement; RCA, right coronary artery; LCA, left coronary artery; THV, transcatheter heart valve; CV, CoreValve; ANeo2, Acurate Neo2; Bic., bicommissural; Tric., tricommissural.

Selective CA of both coronary ostia was possible in all patients before THV implantation. Table 3 summarizes the data on CA after TAVR in fluoroscopically oriented THVs. Post-TAVR combined selective cannulation of both RCA and LCA was achieved in 18 (62.1%) cases, without a significant difference between LCA (82.8%) and RCA (75.9%, P = 0.74) (Figure 4, top panel). Selective RCA cannulation was achieved in 15 (78.9%) of 19 patients with an aligned Evolut Pro/Pro + and in 9 (90%) of 10 patients who received an aligned Acurate Neo2. Selective LCA engagement was possible in 13 (68.9%) of 19 patients who received an aligned Evolut Pro/Pro + and in 9 (90%) of 10 patients with an aligned Acurate Neo2 THV. In all cases, the diagnostic catheter engaged the coronary ostium across the THV stent frame without passing through the virtual space between the THV frame and aortic wall. To note, the absolute rates of selective CA for RCA, LCA, and combined RCA/LCA were higher in the Acurate Neo2 vs. Evolut Pro/Pro + THV and in the tricommissural vs. bicommissural groups, although the differences were not statistically significant.

**TABLE 3:** Procedural results.

Overall population	Overall (*n* = 34)	CoreValve Evolut (*n* = 23)	Acurate Neo2 (*n* = 11)	*P* value	Bicommissural type (*n* = 23)	Tricommissural type (*n* = 11)	*P* value
THV type		−	−	−			
CoreValve Evolut Pro/Pro +	23 (67.6)				17 (73.9)	6 (54.5)	1.00
Acurate Neo2	11 (32.4)				6 (26.1)	5 (45.5)	
TF approach	34 (100)	23 (100)	11 (100)	−	23 (100)	11 (100)	−
Pre-dilatation	34 (100)	23 (100)	11 (100)	−	23 (100)	11 (100)	−
Post-dilatation	12 (35)	7 (30.4)	− (45.5)	0.46	8 (34.8)	4 (36.4)	1.0
Supra-annular THV sizing	20 (59)	15 (65.2)	5 (45.5)	0.46	14 (60.9)	6 (54.5)	1.0
Procedural success	34 (100)	23 (100)	11 (100	−	23 (100)	11 (100	−
In-hospital death	0 (0)	0 (0)	0 (0)	NA	0 (0)	0 (0)	NA
Coronary obstruction	0 (0)	0 (0)	0 (0)	NA	0 (0)	0 (0)	NA
Successful orientation	29 (85.3)	19 (82.6)	10 (90.9)	1.00	21 (91.3)	8 (72.7)	1.00
CV orientation type*	−		−	−			
OC		0 (0)			0	0	−
CB		0 (0)			0	0	
CF		19 (82.6)			15/17	4/6	
IC		4 (17.4)			2/17	2/6	
ANeo2 orientation type*	−	−		−			
FSS/RCC-LCC			10 (90.9)		6/6	4/5	−
FSS/non-RCC-LCC			1 (9.1)		0	1/5	

**Oriented THV**	**Overall** **(*n* = 29)**	**CoreValve Evolut** **(*n* = 19)**	**Acurate Neo2** **(*n* = 10)**	***P* value**	**Bicommissural type** **(*n* = 21)**	**Tricommissural type** **(*n* = 8)**	***P* value**

Oriented THV type				−			1.00
CoreValve Evolut	19 (65.5)	19 (100)	−		15 (71.4)	4 (50)	
Acurate Neo2	10 (34.5)	−	10 (100)		6 (28.6)	4 (50)	
Coronary access – RCA				1.00			1.00
Selective	24 (82.8)	15 (78.9)	9 (90)		17 (81.0)	7 (87.5)	
Sub-selective	4 (13.8)	3 (15.8)	1 (10)		3 (14.3)	1 (12.5)	
Unfeasible	1 (3.4)	1 (5.3)	0 (0)		1 (4.8)	0 (0)	
Coronary access – LCA				0.59			0.74
Selective	22 (75.9)	13 (68.4)	9 (90)		15 (71.4)	7 (87.5)	
Sub-selective	6 (20.7)	5 (26.3)	1 (10)		5 (23.8)	1 (12.5)	
Unfeasible	1 (3.4)	1 (5.3)	0 (0)		1 (4.8)	0 (0)	
Coronary access selective – RCA/LCA	18 (62.1)	10 (52.6)	8 (80.0)	0.23	11 (52.4)	7 (87.5)	0.11
**Coronary access feasible**							
Successful catheter for RCA				−	−	−	−
JR 4	−	16 (88.8)	10 (100)				
AL 1 or 2		2 (11.2)	0 (0)				
Successful catheter for LCA	−			−	−	−	−
JL 3.5 or 4		18 (100)	9 (90)				
XB 3.5 or 4		0 (0)	1 (10)				

Values are n (%). THV, transcatheter heart valve; TF, trans-femoral; CV, CoreValve; OC, outer curve; CB, center back; CF, center front; IC, inner curve; FSS, free stent strut; RCC, right coronary cusp; LCC, left coronary cusp; RCA, right coronary artery; LCA, left coronary artery; JR, Judkins Right; AL, Amplatz Left; JL, Judkins Left; XB, Extra Backup.

*At final check in cusp overlap view.

### Post-transcatheter aortic valve replacement computed tomography

Post-TAVR CT analysis was conducted in the 29 patients with successful fluoroscopic THV orientation. Results of post-procedural CT are listed in Table 4. Supplementary Figures 2, 3 depict the distribution of the variables measured at post-TAVR CT scan according to THV and BAV subgroups, respectively. Observed THV alignment was optimal in 20 (69%) cases, while misalignment was mild in 5 (17.2%), moderate in 2 (6.9%) and severe in 2 (6.9%) cases, without any significant difference between THV nor BAV type subgroups (Figure 4, middle panel). Optimal neo-commissural alignment with respect to both coronaries was observed in 13 (44.8%) cases, without significant difference among THV subgroups (*P* = 0.43). Moderate/severe overlap with the coronaries was seen in 13.7% for the RCA and 44.8% for the LCA (*P* = 0.019) (Figure 4, bottom panel). Twelve (70.6%) of the 17 cases of non-optimal coronary overlap involved the neo-commissures corresponding to native NCC/RCC and NCC/LCC commissures. Overall, median eccentricity index of THV expansion was 17.4%. Moderate or severe THV/LCA overlap was numerically more common in case of asymmetric (i.e., THV eccentricity index > 10%) THV expansion (52.6 vs. 36.4%). Median angle deviation between the raphe and the C-tab or the commissural post corresponding to the FSS was 11.1° [0.6, 49.1]. The Acurate Neo 2 showed a lower misalignment as compared with the Evolut Pro/Pro+ (median angle deviation 7.8° vs. 13.8°), although the difference did not reach statistical significance (*P* = 0.08). Supplementary Figure 4 and Supplementary Tables 1–2 report the rates of THV/coronary overlap and CA according to THV alignment with respect to the raphe. Supplementary Tables 3, 4 report the rates of CA according to THV/coronary overlap. To note, moderate/severe THV misalignment had a significant negative impact on the likelihood of optimal THV/LCA overlap and feasibility of selective CA with regards to the LCA or combined RCA/LCA. Similarly, moderate/severe THV coronary overlap had a significant negative impact on the likelihood of successful CA.

**TABLE 4:** Post-procedural CT analysis –Oriented THVs.

	Overall *n* = 29	CoreValve Evolut *n* = 19	Acurate Neo2 *n* = 10	*P* value	Bicommissural type *n* = 21	Tricommissural type *n* = 8	*P* value
Angle RCA/C-tab or FSS,°	66.5 (57.2-70.7)	65.0 (37.0-96.4)	67.3 (48.0-109.1)	0.71	68.9 (42.0-109.1)	55.4 (37.0-75.3)	**0.025**
Angle RCA/THV NCC-RCC com,°	55.7 (46.7-66.7)	55.6 (24.4-89.7)	63.1 (8.8-88.3)	1.00	54.5 (8.8-88.3)	66.4 (46.7-89.7)	**0.045**
Angle LCA/C-tab or FSS,°	70.4 (56.4-77.3)	70.2 (12.4-122.6)	73.7 (40.9-96.5)	0.96	69.6 (12.4-122.6)	74.4 (57.6-107.1)	0.35
Angle LCA/THV NCC-LCC com,°	45.3 (33.8-66.9)	45.3 (2.2-82.7)	41.2 (15.2-81.1)	0.75	49.7 (2.2-82.7)	43.7 (13.9-55.5)	0.31
Angle THV C-tab or FSS/NCC-RCC com,°	123.9 (117.9-126.4)	123.9 (110.5-137.6)	123.2 (108.8-134.3)	0.82	123.9 (108.8-137.6)	123.3 (116.8-134.3)	0.77
Angle THV C-tab or FSS/NCC-LCC com,°	119.3 (111.6-122.9)	119.3 (90.8-132.8)	115.6 (98.8-126.6)	0.58	120.9 (90.8-132.8)	117.2 (98.8-26.1)	0.41
Angle THV NCC-RCC/NCC-LCC com,°	120.3 (111.3-124.6)	120.3 (100.9-138.4)	122.0 (102.8-140.6)	0.71	120.3 (100.9-138.4)	121.3 (109.4-140.6)	0.96
Angle deviation raphe/C-tab or FSS,°	11.0 (0.6-49.1)	13.8 (3.8-49.1)	7.8 (0.6-26.8)	0.08	11.8 (1.8-49.2)	7.8 (0.6-42.7)	0.70
Distance THV-RCA, mm	5.7 (4.5-6.9)	5.7 (3.1-10.1)	5.5 (3.4-9.5)	0.68	5.7 (3.4-8.6)	5.7 (3.1-10.1)	0.26
Distance THV-LCA, mm	5.9 (4.8-8.1)	7.0 (4.2-13.5)	4.9 (3.3-8.3)	**0.004**	5.9 (3.4-13.5)	5.6 (3.3-11.2)	0.66
Eccentricity Index,%	17.4 (4.8-20.5)	11.6 (1.0-30.2)	19.4 (4.6-42.8)	**0.005**	17.4 (1.0-39.9)	15.7 (3.1-42.8)	0.66
THV implantation depth, mm	4.65 (3.95-5.4)	4.75 (4.0-5.45)	4.60 (3.95-4.80)	0.74	4.10 (3.95-5.20)	4.75 (4.10-5.45)	0.43
THV alignment Optimally aligned Mildly misaligned Moderately misaligned Severely misaligned	20 (69) 5 (17.2) 2 (6.9) 2 (6.9)	12 (63.2) 3 (15.8) 2 (10.5) 2 (10.5)	8 (80) 2 (20) 0 (0) 0 (0)	0.69	15 (71.4) 3 (14.3) 1 (4.8) 2 (9.5)	5 (62.5) 2 (25) 1 (12.5) 0 (0)	0.64
Coronary overlap – RCA Optimal Moderate Severe	25 (86.2) 3 (10.3) 1 (3.4)	17 (89.5) 2 (10.5) 0 (0)	8 (80) 1 (10) 1 (10)	0.59 1.00 0.35	18 (85.7) 2 (9.5) 1 (4.8)	7 (87.5) 1 (12.5) 0 (0)	1.00 1.00 1.00
Coronary overlap – LCA Optimal Moderate Severe	16 (55.2) 8 (27.6) 5 (17.2)	11 (57.9) 4 (21.1) 4 (21.1)	5 (50) 4 (40) 1 (10)	0.71 0.39 0.63	11 (52.4) 6 (28.6) 4 (19)	5 (62.5) 2 (25) 1 (12.5)	0.70 1.00 1.00
Optimal coronary overlap – RCA/LCA	13 (44.8)	10 (52.6)	3 (30)	0.43	8 (38.1)	5 (62.5)	0.41

Values are *n* (%), or median (range). CT, computed tomography; THV, transcatheter heart valve; RCA, right coronary artery; LCA, left coronary artery; FSS, free stent strut; NCC, non-coronary cusp; RCC, right coronary cusp; LCC, left coronary cusp; com, commissure. Bold values are those statistically significant.

*Evolut Pro/Pro* +. Median angle deviation between the raphe and C-tab was 13.8° [3.8, 49.1], with an observed rate of optimal THV alignment of 63.2% (12/19 implants). Overlap between the RCA and the C-tab or commissural post corresponding to the native NCC-RCC commissure was optimal in 17 (89.5%), moderate in 2 (10.5%) cases. Overlap between the LCA and the C-tab or commissural post corresponding to the native NCC-LCC commissure was optimal in 11 (58%), moderate in 4 (21%) cases, and severe in 4 (21%) cases. Median distance of the THV frame from the coronaries was 5.7 mm [3.1, 10.1 mm] for the RCA and 7.0 mm [4.2, 13.5] for the LCA. A THV eccentricity index < 10% was found in 6 patients (31.6%), with a median value of 11.6% [1.0, 30.2%].

*Acurate Neo2.* Median angle deviation between the raphe and the commissural post corresponding to the FSS was 7.8° [0.6, 26.8°], with an observed rate of optimal THV alignment of 80% (8/10 implants). Overlap between the RCA and the commissural post corresponding to the FSS or commissural post corresponding to the native NCC-RCC commissure was optimal in 8 (80%), moderate in 1 (10%), and severe in 1 (10%) case. Overlap between the LCA and the commissural post corresponding to the FSS or commissural post corresponding to the native NCC-LCC commissure was optimal in 5 (50%), moderate in 4 (40%), and severe in 1 (10%) case. Median distance of the THV frame from the coronaries was 5.5 mm [3.4, 9.5 mm] for the RCA and 4.9 mm [3.3, 8.3 mm] for the LCA. A THV eccentricity index < 10% was found in 2 patients (20%), with a median value of 19.4% [4.6, 42.8%], which was significantly higher compared with the Evolut group (*P* = 0.005).

**FIGURE 5:**
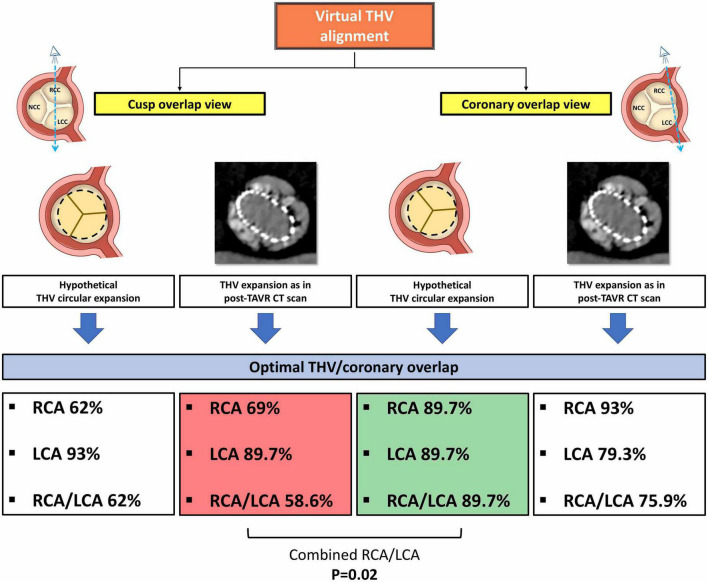
Illustration of the virtual THV alignment simulation with rates of optimal THV/coronary overlap. See the description in the main text. THV, transcatheter heart valve; RCA, right coronary artery; LCA, left coronary artery.

*Virtual THV commissural alignment.* Supplementary Tables 5–8 present the rates of coronary overlap with different virtual scenarios of THV alignment. Given a more pronounced eccentricity of the RCA, when the THV was aligned with the raphe, the rates of non-optimal RCA overlap were higher than for the LCA (virtual scenarios #3 and #4). To note, a THV alignment aimed at the bisector of the coronary ostia angle (i.e., “coronary overlap”) is expected to produce an optimal overlap between THV and both the coronaries in almost 90% of cases, assuming a hypothetical circular valve expansion (Table 5 and Figure 5).

**TABLE 5:** Coronary overlap and THVs alignment scenarios.

	Optimal coronary overlap – RCA *n* = 29	*P* value	Optimal coronary overlap – LCA *n* = 29	*P* value	Optimal coronary overlap – RCA/LCA *n* = 29	*P* value
THV orientation model a. Observed b. Virtual scenario #1	25 (86.2) 26 (89.7)	1.00	16 (55.2) 26 (89.7)	**0.007**	13 (44.8) 26 (89.7)	**0.006**
THV orientation model a. Observed b. Virtual scenario #2	25 (86.2) 27 (93)	0.67	16 (55.2) 23 (79.3)	0.09	13 (44.8) 22 (75.9)	**0.03**
THV orientation model a. Virtual scenario #1 b. Virtual scenario #2	26 (89.7) 27 (93)	1.00	26 (89.7) 23 (79.3)	0.47	26 (89.7) 22 (75.9)	0.30
THV orientation model a. Observed b. Virtual scenario #3	25 (86.2) 18 (62)	0.07	16 (55.2) 27 (93)	**0.002**	13 (44.8) 18 (62)	0.29
THV orientation model a. Virtual scenario #1 b. Virtual scenario #3	26 (89.7) 18 (62)	**0.03**	26 (89.7) 27 (93)	1.00	26 (89.7) 18 (62)	**0.03**
THV orientation model a. Virtual scenario #2 b. Virtual scenario #3	27 (93) 18 (62)	**0.001**	23 (79.3) 27 (93)	0.25	22 (75.9) 18 (62)	0.39
THV orientation model a. Observed b. Virtual scenario #4	25 (86.2) 20 (69)	0.21	16 (55.2) 26 (89.7)	**0.007**	13 (44.8) 17 (58.6)	0.43
THV orientation model a. Virtual scenario #1 b. Virtual scenario #4	26 (89.7) 20 (69)	0.10	26 (89.7) 26 (89.7)	1.00	26 (89.7) 17 (58.6)	**0.02**
THV orientation model a. Virtual scenario #2 b. Virtual scenario #4	27 (93) 20 (69)	0.09	23 (79.3) 26 (89.7)	0.47	22 (75.9) 17 (58.6)	0.26
THV orientation model a. Virtual scenario #3 b. Virtual scenario #4	18 (62) 20 (69)	0.78	27 (93) 26 (89.7)	1.00	18 (62) 17 (58.6)	1.00

Values are *n* (%). THV, transcatheter heart valve; RCA, right coronary artery; LCA, left coronary artery. Bold values are those statistically significant.

## Discussion

The DA VINCI study is the first prospectively exploring how standard THV alignment techniques perform in terms of THV/coronary overlap in patients with BAV undergoing TAVR. The main findings are: (1) type 1 BAV represents a highly variable anatomical setting characterized by cusp asymmetry and coronary eccentricity; (2) a standard commissural alignment technique based on the cusp overlap view is associated with less favorable THV alignment and higher rates of moderate/severe neo-commissure overlap with the coronary ostia; as a result, rates of selective coronary cannulation after TAVR are lower than in previous reports on TAV; (3) according to our virtual model, a THV alignment technique based on the coronary ostia overlap view might be more effective in preserving CA in selected BAV raphe-type patients.

BAV with raphe represents an extremely variable anatomical setting, from tricommissural BAV, which most closely resembles a TAV and carries commissural angles of almost 120°, to bicommissural BAV, which carries different cusp sizes and inter-commissural angles (11, 13, 17). Moreover, BAV anatomy is characterized by atypical location of coronary ostia, which can be highly eccentric and closer to the non-coronary commissure than in TAV (11). In a recent study by Wang et al. (12), severe cusp asymmetry was found in 52% of BAV patients, whilst it was only 2.5% in TAV. Moreover, the RCA was found to be more eccentric than the LCA with respect to the raphe (12). Consistently, in our study a moderate or severe cusp asymmetry was encountered in about half of BAV patients, particularly in those with bicommissural BAV type. Notably, in our population the prevalence of moderate/severe coronary eccentricity was more than three-fold as compared to the aforementioned study.

As current alignment techniques are largely dependent on the symmetry of TAV anatomy (7), characterized by similar angles among the 3 commissures as well as by an equal distance between coronary ostia and both commissures, little is known about their efficacy and reproducibility in the setting of BAV. The current paper is the first to prospectively evaluate the impact of BAV anatomical variability on final THV alignment. By our data, a standard THV orientation technique based on the cusp overlap view resulted in optimal THV orientation/mild misalignment with the raphe in 86.2% of cases. Nevertheless, an optimal THV/coronary ostium overlap - as assessed by post-TAVR CT - was found only in two-thirds of our BAV population. These results seem to be at odds with the conclusions of previously exploratory studies (12), which suggested that a standard cusp overlap view would be an effective approach to implant one THV commissure in the center between both coronary ostia in the vast majority of BAV patients. As expected, our rates of optimal THV/coronary overlap in BAV patients were also significantly lower than those reported in previous studies focused on TAV (8, 12). We found that severe neo-commissure overlap was five-times higher for the LCA compared to RCA, despite the lower eccentricity of LCA ostium. We can speculate that the latter finding might be secondary to non-circular THV expansion at the level of maximal raphe protrusion, leading to unequal angle between the neo-commissures. As a matter of fact, a final THV eccentricity < 10% was observed in approximately one-thirds of patients. Therefore, in BAV more than in TAV, the spatial relationship between neo-commissures and coronary ostia may be affected by the unpredictable THV geometrical distortion due to the resistance offered by raphe (20, 21). Whether aggressive post-dilatation could have an impact on THV circularity and, therefore, on symmetry of inter-commissural angles is still debated and should be balanced against the risk of aortic injury, that is higher in BAV anatomy (17). Similarly, THV (under)sizing based on supra-annular approach could theoretically minimize the THV distortion at raphe plane. To this regard, in our study, a raphe-based sizing was adopted in roughly two-thirds of procedures, although this did not seem to translate in a high rate of THV circularity, perhaps due to the presence of heavily calcified raphes. Therefore, in the absence of standardization on how to approach THV sizing in BAV, larger studies are needed to better investigate the potential impact of different sizing methods on final THV expansion and commissural alignment.

In addition, our study is also the first to investigate the feasibility of coronary cannulation after TAVR with intentional THV orientation in a BAV population. This aspect is of utmost importance, as BAV subjects are usually younger and thus more likely to require future coronary interventions (1–3). Selective CA was achieved less frequently than we found in previous TAV series. As a matter of fact, in the ALIGN-ACCESS study the orientation of supra-annular tall-frame THVs in TAV was higher than 85% for both Evolut and Acurate systems, with rates of unfeasible coronary cannulation < 3% when the THV was correctly oriented (9). In the current study, despite a similar favorable THV orientation rate at fluoroscopy, an unfeasible/non-selective CA of at least one coronary was found in 40% of cases. Consistently, optimal combined THV/coronary overlap rate at post-TAVR CT scans was less than 50%. Notably, relative selective CA rate did not significantly differ between the RCA and LCA (P = 0.74), although the neo-commissural overlap was less favorable with the LCA. The fact that the angle deviation was higher than 30° in 5 of the 8 cases of moderate THV/LCA overlap, combined with a higher LCA take-off than RCA, might have mitigated the THV interference with LCA cannulation. Another factor of concern related to CA is the THV implantation depth (9, 22), that is usually higher in BAV than in TAV (11). Notwithstanding, we failed to find a difference in the rate of selective coronary cannulation regardless of implantation depth, likely because of the high coronary take-off (particularly in the bicommissural type) seen in our population. Finally, asymmetric THV expansion in the presence of a raphe between the R-L cusp resulted in increased THV-SoV space in front of the coronary ostia, acting as a favoring factor for CA as compared to TAV (9, 10). The latter situation may be of relevance in case of significant aortic dilation, as in that case the catheter has the space to pass outside the THV stent frame to engage the coronary ostium, making the final orientation of the THV commissural posts less impacting.

Little is known about the customization of THV orientation in BAV. Is coronary ostia overlap view more efficient than cusp overlap view in achieving optimal THV/coronary alignment in BAV patients? What is the interplay between THV alignment and final THV underexpansion, frequently observed in BAV anatomies? (12, 18). The THV alignment technique adopting the cusp-overlap view assumes that the coronary arteries originate from the center of the aortic valve cusp and that the angle between the two native commissures is even, which - as observed in our population - is often not the case in BAV. According to our virtual THV model, coronary ostia overlap vs. a standard cusp overlap alignment would increase optimal THV/coronary overlap rates by 70% and 40% in case of circular and eccentric THV expansion, respectively (Table 5 and Figure 5). However, it should be noted that in the not so infrequent situation in BAV of extremely wide inter-coronary ostia angle (> 180°), even THV orientation using coronary ostia overlap view with neo-commissural angles of 120° apart would inevitably lead to unfavorable THV/coronary overlap. Finally, considering the complex interplay between anatomical and procedural factors that may impact coronary cannulation, if the systematic use of a coronary ostia overlap-based alignment in BAV will traduce in significantly higher rates of selective CA has yet to be proved.

Minimizing the overlap between neo-commissures and coronary ostia is also important for the lifetime strategy of patients with bicuspid aortic stenosis, as they represent a younger population that could often outlive the THV durability, with the eventual need for redo-TAVR (1, 3). Particularly with degenerated supra-annular tall-frame THVs, a moderate/severe THV coronary overlap may hamper the efficacy of BASILICA (bioprosthetic or native aortic scallop intentional laceration to prevent coronary artery obstruction), as the beneficial effect of this technique decreases if the leaflet is lacerated in proximity of the commissural post (23). To note, future research is needed to assess whether a coronary-based vs. a commissure-based THV alignment will provide worse hemodynamic status and THV durability in BAV patients with longer life expectancy (24).

### Study limitations

The present work has the inherent limitations of an observational single-center study without core-lab analysis. Due to the small sample size, patient selection bias could not be excluded and direct comparison between THV type was not possible. The classification of BAV asymmetry (in terms of cusp dimensions and coronary eccentricity) and of THV/coronary overlap is arbitrary but based on previously published reports (6, 12, 23). As less common BAV types (i.e., type 0 and 2) were excluded and all included subjects had BAV with R-L raphe, our findings cannot be generalized to all BAV anatomies. Moreover, broad generalization to other THV platforms not included in the study cannot be undertaken. Although the absolute rate of successful CA was higher with the Acurate Neo2 platform, the potential advantage of the Acurate Neo2 open-cell design as compared to the Evolut Pro/Pro + in case of misalignment remains speculative. We cannot exclude that selective engagement of coronary ostium could be achieved in a higher percentage of patients if further procedural techniques (such as the use of coronary guidewires or guide extension catheters) had been adopted. Finally, as our comparison between different THV alignment techniques was based on virtual THV modeling (thus potentially not accounting for other procedural variables) definitive inference on this argument cannot be drawn. Further studies on this topic are eagerly awaited.

## Conclusion

**FIGURE 6:**
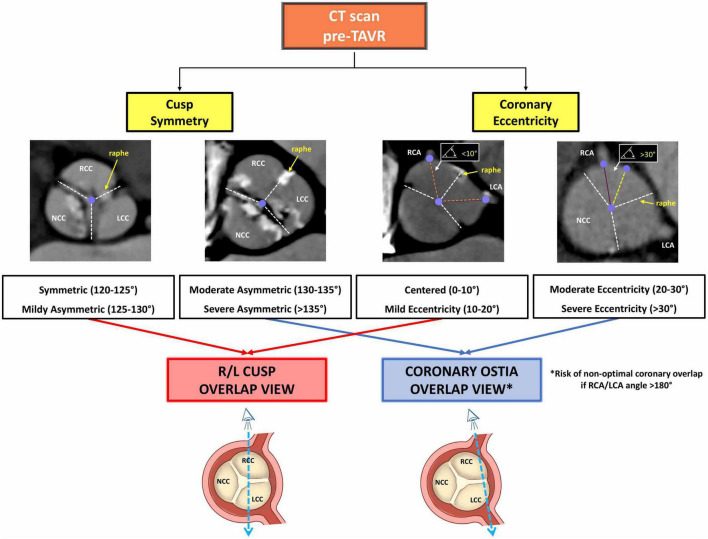
Illustration of the flow algorithm when evaluating pre-procedural CT scan to customize THV alignment in BAV. CT, computed tomography; THV, transcatheter heart valve; BAV, bicuspid aortic valve; TAVR, transcatheter aortic valve replacement; R, right; L, left; RCA, right coronary artery; LCA, left coronary artery.

Bicuspid aortic stenosis has a highly variable anatomy characterized by cusp asymmetry and eccentricity of coronary ostia. Use of conventional commissural alignment techniques in this setting is feasible, although associated with higher rates of THV misalignment and of moderate/severe neo-commissure overlap with the coronary ostia. As a result, rates of selective coronary cannulation after TAVR are lower in BAV as compared to TAV. By our virtual THV modeling, an orientation technique based on the coronary ostia overlap rather than the cusp overlap view seems to be more accurate in minimizing overlap between neo-commissures and coronary ostia, regardless of final THV expansion, in BAV patients and, as such, it should be pursued when anatomy is severely asymmetric (Figure 6). Further studies are needed to validate in larger BAV populations the reproducibility of a customized coronary-overlap THV alignment technique as related to THV/coronary overlap and CA after TAVR.

## Data availability statement

The raw data supporting the conclusions of this article will be made available by the authors, without undue reservation.

## Ethics statement

The studies involving human participants were reviewed and approved by IRB of the University Hospital of Padua. The patients/participants provided their written informed consent to participate in this study.

## Author contributions

TF, LNF, FC, SC, and GT participated to the conception, design, analysis, interpretation of data, and drafting of the manuscript. All authors contributed providing a critical revision for important intellectual content, giving the final approval of the submitted text, agreed to be accountable for all aspects of the work in ensuring that questions related to the accuracy or integrity of any part of the work are appropriately investigated and resolved, and have contributed significantly to the submitted work.

## Supplementary material

Supplementary Figure 1 Illustration of the distribution (box plot with inter-quartile ranges) of the variables measured at basal CT scan according to BAV types. TAVR, transcatheter aortic valve replacement; CT, computed tomography; BAV, bicuspid aortic valve; NCC, non-coronary cusp; RCC, right coronary cusp; LCC, left coronary cusp; RCA, right coronary artery; LCA, left coronary artery; Bic., bicommissural; Tric., tricommissural.

Supplementary Figure 2 Illustration of the distribution (box plot with inter-quartile ranges) of the variables measured at post-procedural CT scan according to THV subgroups. TAVR, transcatheter aortic valve replacement; CT, computed tomography; THV, transcatheter heart valve; NCC, non-coronary cusp; RCC, right coronary cusp; LCC, left coronary cusp; RCA, right coronary artery; LCA, left coronary artery; FSS, free stent strut; CV, CoreValve; ANeo2, Acurate Neo2.

Supplementary Figure 3 Illustration of the distribution (box plot with inter-quartile ranges) of the variables measured at post-procedural CT scan according to BAV subgroups. TAVR, transcatheter aortic valve replacement; CT, computed tomography; BAV, bicuspid aortic valve; CT, computed tomography; THV, transcatheter heart valve; NCC, non-coronary cusp; RCC, right coronary cusp; LCC, left coronary cusp; RCA, right coronary artery; LCA, left coronary artery; FSS, free stent strut; Bic., bicommissural; Tric., tricommissural.

Supplementary Figure 4 Illustration of the rates of neo-commissural overlap according to degree of THV alignment as respect to raphe in the 29 patients with fluoroscopically oriented THV. THV, transcatheter heart valve; RCA, right coronary artery; LCA, left coronary artery.
