# Supplementary figures and images for: Definition of trAnscatheter heart Valve orIeNtation in biCuspId aortic valve: The DA VINCI pilot study
Source: Front Cardiovasc Med. 2022 Dec 12;9:1056496. doi: 10.3389/fcvm.2022.1056496 (PMC9790995; doi:10.3389/fcvm.2022.1056496)

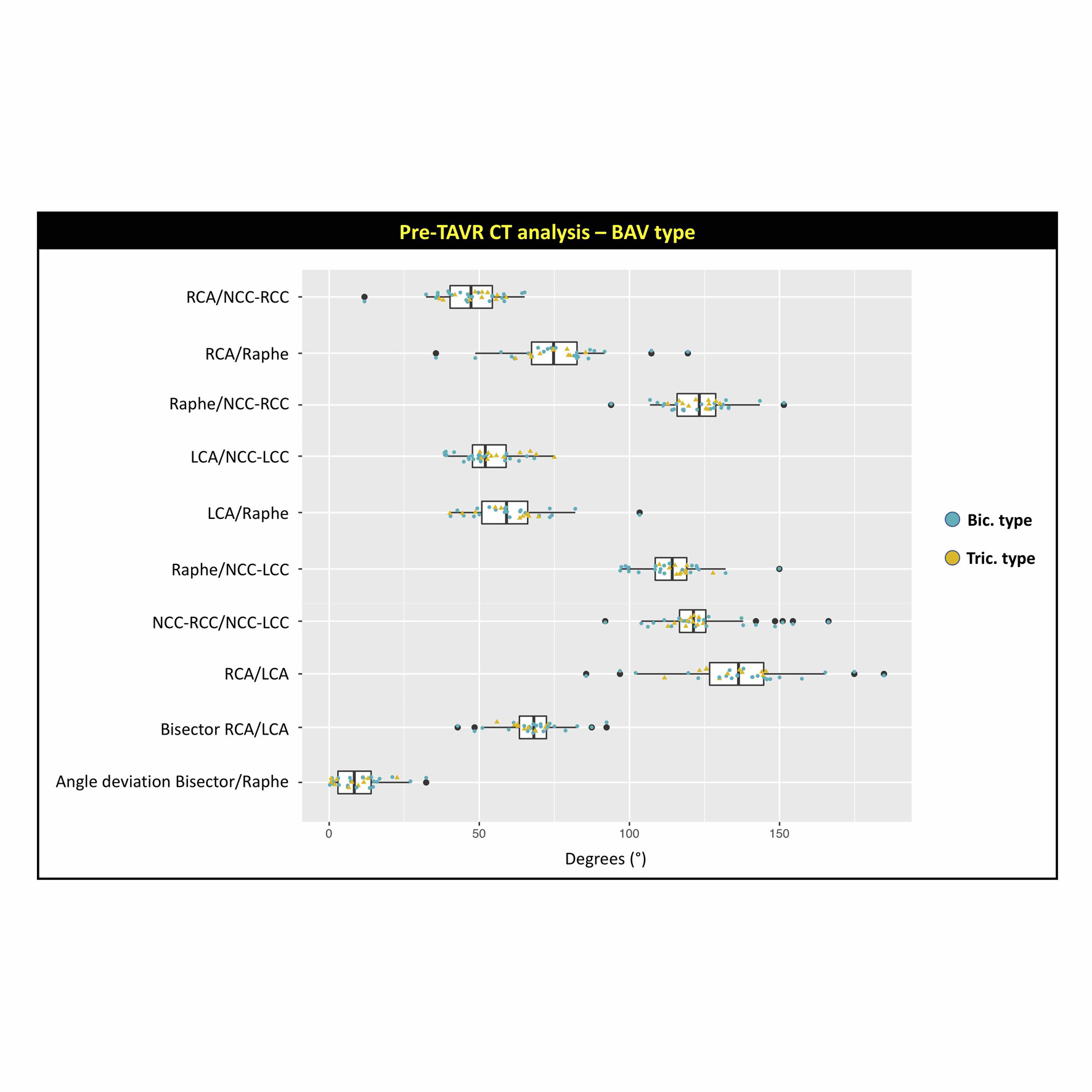

Supplement: Supplementary Figure 1 — Illustration of the distribution (box plot with inter-quartile ranges) of the variables measured at basal CT scan according to BAV types. TAVR, transcatheter aortic valve replacement; CT, computed tomography; BAV, bicuspid aortic valve; NCC, non-coronary cusp; RCC, right coronary cusp; LCC, left coronary cusp; RCA, right coronary artery; LCA, left coronary artery; Bic., bicommissural; Tric., tricommissural. [file Image_1.JPEG]

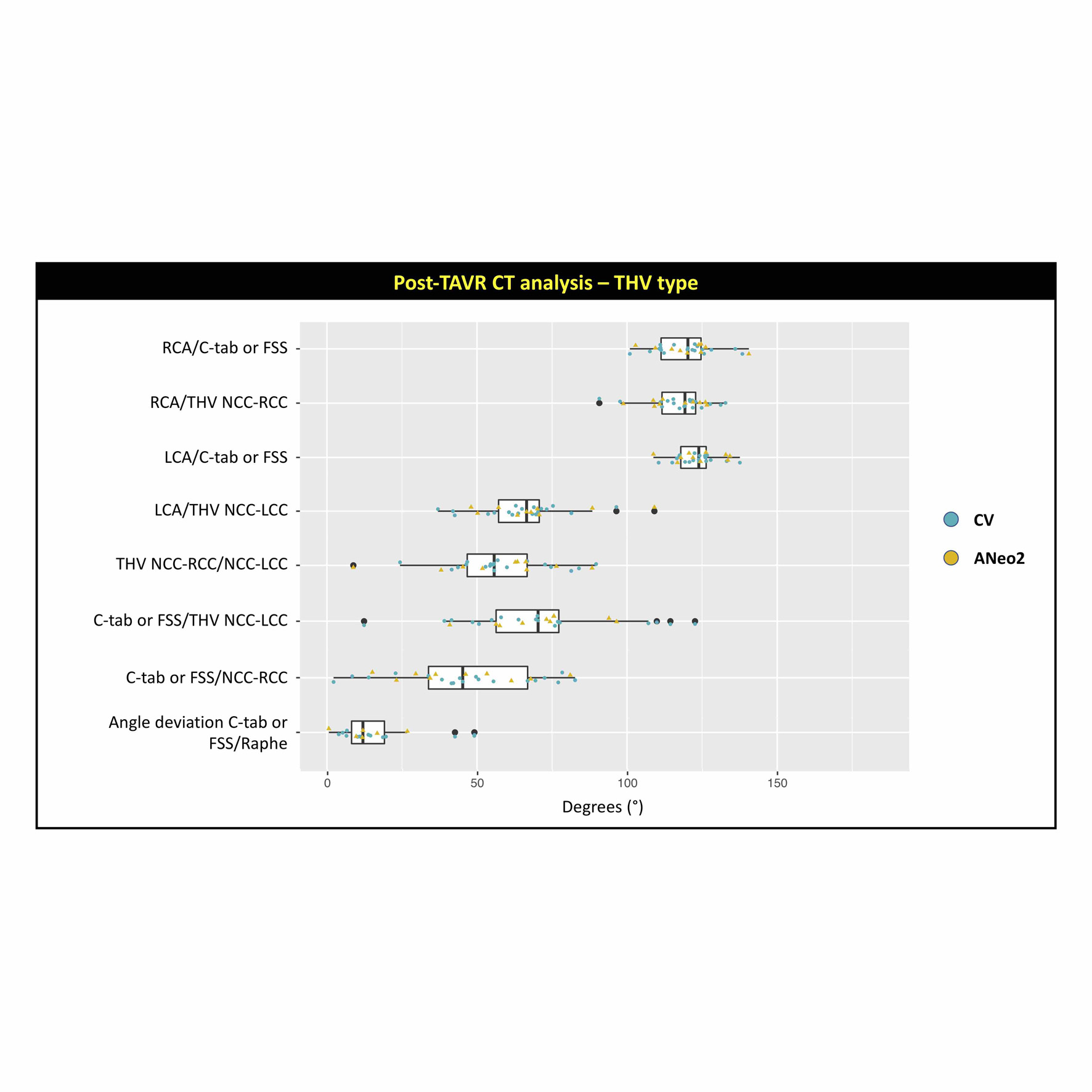

Supplement: Supplementary Figure 2 — Illustration of the distribution (box plot with inter-quartile ranges) of the variables measured at post-procedural CT scan according to THV subgroups. TAVR, transcatheter aortic valve replacement; CT, computed tomography; THV, transcatheter heart valve; NCC, non-coronary cusp; RCC, right coronary cusp; LCC, left coronary cusp; RCA, right coronary artery; LCA, left coronary artery; FSS, free stent strut; CV, CoreValve; ANeo2, Acurate Neo2. [file Image_2.JPEG]

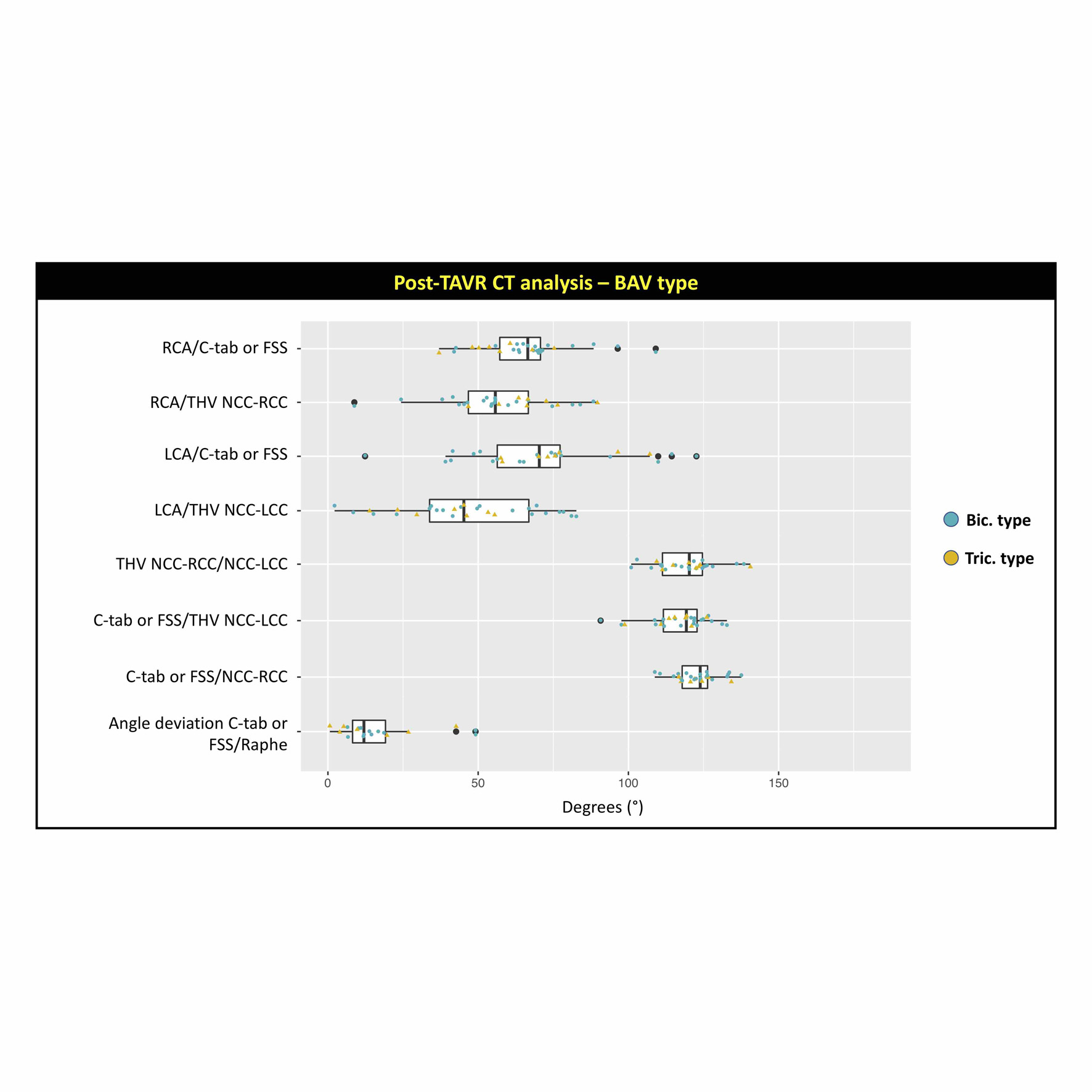

Supplement: Supplementary Figure 3 — Illustration of the distribution (box plot with inter-quartile ranges) of the variables measured at post-procedural CT scan according to BAV subgroups. TAVR, transcatheter aortic valve replacement; CT, computed tomography; BAV, bicuspid aortic valve; CT, computed tomography; THV, transcatheter heart valve; NCC, non-coronary cusp; RCC, right coronary cusp; LCC, left coronary cusp; RCA, right coronary artery; LCA, left coronary artery; FSS, free stent strut; Bic., bicommissural; Tric., tricommissural. [file Image_3.JPEG]

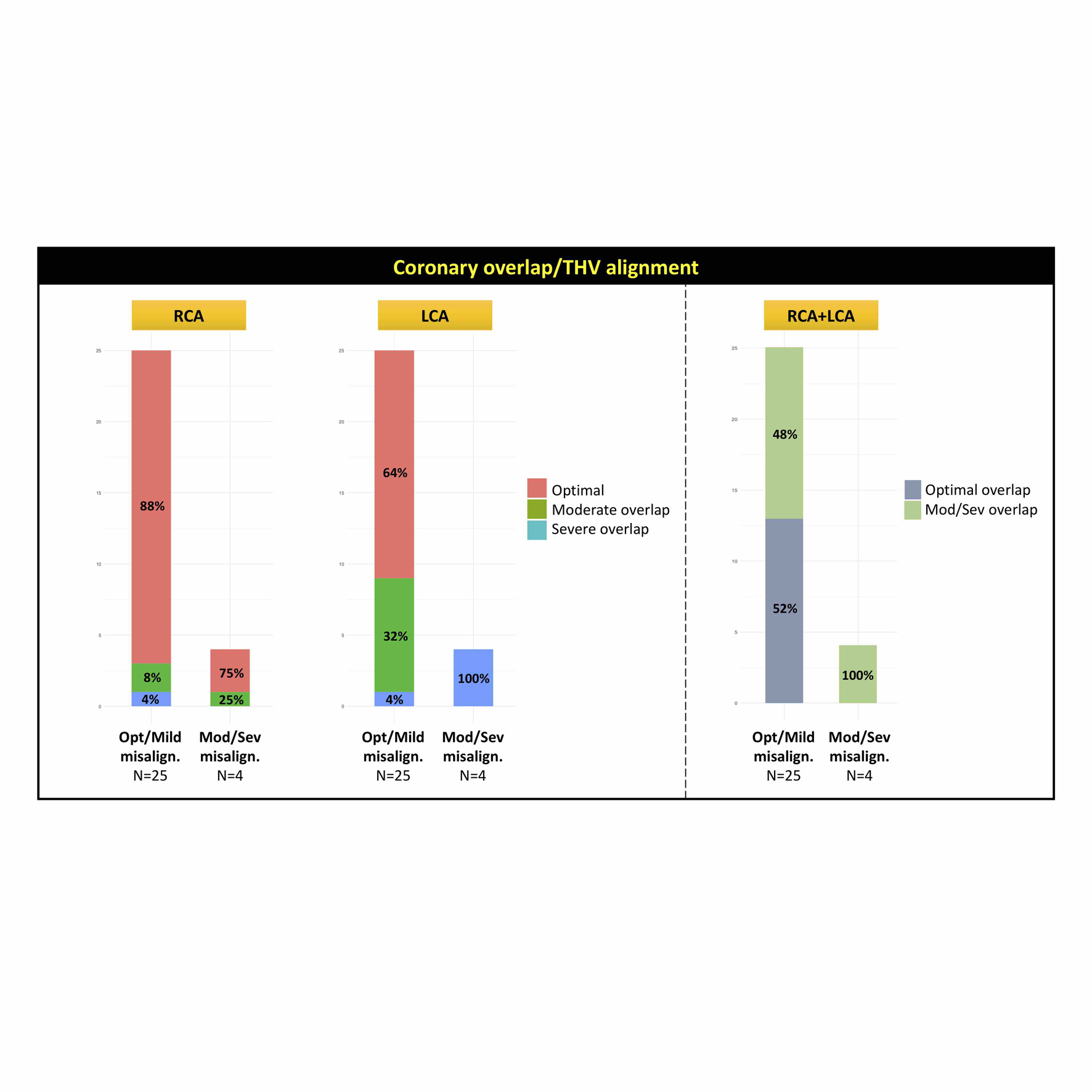

Supplement: Supplementary Figure 4 — Illustration of the rates of neo-commissural overlap according to degree of THV alignment as respect to raphe in the 29 patients with fluoroscopically oriented THV. THV, transcatheter heart valve; RCA, right coronary artery; LCA, left coronary artery. [file Image_4.JPEG]
