# Supplementary material for: Definition of trAnscatheter heart Valve orIeNtation in biCuspId aortic valve: The DA VINCI pilot study
Source: Front Cardiovasc Med. 2022 Dec 12;9:1056496. doi: 10.3389/fcvm.2022.1056496 (PMC9790995; doi:10.3389/fcvm.2022.1056496)
Supplement: Supplementary file 5 [file Data_Sheet_1.docx]

**SUPPLEMENTAL TABLES**

**Supplemental Table 1.** Coronary overlap and observed THVs alignment.

|  | **Overall**  n=29 | **Optimal**  **THV alignment**  n=20 | **Mild**  **THV misalignment**  n=5 | **Moderate**  **THV misalignment**  n=2 | **Severe**  **THV misalignment**  n=2 | **P value** |
| --- | --- | --- | --- | --- | --- | --- |
| Coronary overlap – RCA   1. Optimal 2. Moderate 3. Severe | 25 (86.2)  3 (10.3)  1 (3.4) | 17 (85)  2 (10)  1 (5) | 5 (100)  0 (0)  0 (0) | 1 (50)  1 (50)  0 (0) | 2 (100)  0 (0)  0 (0) | 0.34  0.24  0.93 |
| Coronary overlap – LCA   1. Optimal 2. Moderate 3. Severe | 16 (55.2)  8 (27.6)  5 (17.2) | 14 (70)  5 (25)  1 (5) | 2 (40)  3 (60)  0 (0) | 0 (0)  0 (0)  2 (100) | 0 (0)  0 (0)  2 (100) | 0.67  0.24  **<0.001** |
| Optimal coronary overlap – RCA/LCA | 13 (44.8) | 11 (55) | 2 (40) | 0 (0) | 0 (0) | 0.25 |

Values are n (%)

THV, transcatheter heart valve; RCA, right coronary artery; LCA, left coronary artery.

**Supplemental Table 2.** Coronary access and observed THVs alignment.

|  | **Overall**  n=29 | **Optimal**  **THV alignment**  n=20 | **Mild**  **THV misalignment**  n=5 | **Moderate**  **THV misalignment**  n=2 | **Severe**  **THV misalignment**  n=2 | **P value** |
| --- | --- | --- | --- | --- | --- | --- |
| Coronary access – RCA   1. Selective 2. Sub-selective 3. Unfeasible | 24 (82.8)  4 (13.8)  1 (3.4) | 16 (80)  3 (15)  1 (5) | 5 (100)  0 (0)  0 (0) | 1 (50)  1 (50)  0 (0) | 2 (100)  0 (0)  0 (0) | 0.69 |
| Coronary access – LCA   1. Selective 2. Sub-selective 3. Unfeasible | 22 (75.9)  6 (20.7)  1 (3.4) | 17 (85)  3 (15)  0 (0) | 5 (100)  0 (0)  0 (0) | 0 (0)  2 (100)  0 (0) | 0 (0)  1 (50)  1 (50) | **<0.001** |
| Coronary access selective – RCA/LCA | 18 (62.1) | 13 (65) | 5 (100) | 0 (0) | 0 (0) | **0.02** |

Values are n (%)

THV, transcatheter heart valve; RCA, right coronary artery; LCA, left coronary artery.

**Supplemental Table 3.** Coronary access and observed overlap for RCA and LCA.

|  | **Optimal overlap**  n=25 | **Moderate/Severe overlap**  n=4 | **P value** |
| --- | --- | --- | --- |
| Coronary access – RCA   1. Selective 2. Sub-selective/Unfeasible | 23 (92)  2 (8) | 1 (25)  3 (75) | **<0.001** |
|  | **Optimal overlap**  n=16 | **Moderate/Severe overlap**  n=13 | **P value** |
| Coronary access – LCA   1. Selective 2. Sub-selective/Unfeasible | 15 (93.7)  1 (6.3) | 7 (53.8)  6 (46.2) | **0.024** |

Values are n (%)

RCA, right coronary artery; LCA, left coronary artery.

**Supplemental Table 4.** Coronary access and observed overlap for RCA/LCA.

|  | **Optimal Overlap**  **RCA-LCA**  n=13 | **Non optimal Overlap**  **RCA-LCA**  n=16 | **P value** |
| --- | --- | --- | --- |
| Coronary access selective – RCA/LCA | 11 | 7 | 0.052 |

Values are n (%)

RCA, right coronary artery; LCA, left coronary artery.

**Supplemental Table 5.** THV alignment – virtual scenario #1 (see methods).

|  | **Overall**  n=29 | **CoreValve Evolut**  n=19 | **Acurate Neo2**  n=10 | **P value** | **Bicommissural type**  n=21 | **Tricommissural type**  n=8 | **P value** |
| --- | --- | --- | --- | --- | --- | --- | --- |
| Angle RCA/C-tab or FSS, ° | 69.0 (65.0-72.7) | 68.8 (42.8-92.4) | 71.4 (61.7-78.7) | 0.44 | 70.4 (42.8-92.4) | 67.6 (55.9-72.7) | 0.38 |
| Angle RCA/THV NCC-RCC com, ° | 51.0 (47.3-55.0) | 51.2 (27.6-77.2) | 48.7 (41.3-58.3) | 0.44 | 49.6 (27.6-77.2) | 52.4 (47.3-64.1) | 0.38 |
| Angle LCA/C-tab or FSS, ° | 69.0 (65.0-72.7) | 68.8 (42.8-92.4) | 71.4 (61.7-78.7) | 0.44 | 70.4 (42.8-92.4) | 67.6 (55.9-72.7) | 0.38 |
| Angle LCA/THV NCC-LCC com, ° | 51.0 (47.3-55.0) | 51.2 (27.6-77.2) | 48.7 (41.3-58.3) | 0.44 | 49.6 (27.6-77.2) | 52.4 (47.3-64.1) | 0.38 |
| Coronary overlap – RCA   1. Optimal 2. Moderate 3. Severe | 26 (89.7)  3 (10.3)  0 (0) | 16 (84.2)  3 (15.8)  0 (0) | 10 (100)  0 (0)  0 (0) | 0.53  0.53  NA | 18 (85.7)  3 (14.3)  0 (0) | 8 (100)  0 (0)  0 (0) | 0.54  0.54  NA |
| Coronary overlap – LCA   1. Optimal 2. Moderate 3. Severe | 26 (89.7)  3 (10.3)  0 (0) | 16 (84.2)  3 (15.8)  0 (0) | 10 (100)  0 (0)  0 (0) | 0.53  0.53  NA | 18 (85.7)  3 (14.3)  0 (0) | 8 (100  0 (0)  0 (0) | 0.54  0.54  NA |
| Optimal coronary overlap – RCA/LCA | 26 (89.7) | 16 (84.2) | 10 (100) | 0.53 | 18 (85.7) | 8 (100) | 0.54 |

Values are n (%), or median (range)

THV, transcatheter heart valve; RCA, right coronary artery; LCA, left coronary artery; FSS, free stent strut; NCC, non-coronary cusp; RCC, right coronary cusp; LCC, left coronary cusp; com, commissure.

**Supplemental Table 6.** THV alignment – virtual scenario #2 (see methods).

|  | **Overall**  n=29 | **CoreValve Evolut**  n=19 | **Acurate Neo2**  n=10 | **P value** | **Bicommissural type**  n=21 | **Tricommissural type**  n=8 | **P value** |
| --- | --- | --- | --- | --- | --- | --- | --- |
| Angle RCA/C-tab or FSS, ° | 69.0 (65.0-72.7) | 68.8 (42.8-92.4) | 71.4 (61.7-78.7) | 0.44 | 70.4 (42.8-92.4) | 67.6 (55.9-72.7) | 0.38 |
| Angle RCA/THV NCC-RCC com, ° | 52.2 (47.5-61.3) | 51.8 (31.9-90.4) | 53.3 (40.8-71.5) | 0.85 | 50.7 (31.9-90.4) | 54.5 (48.8-71.5) | 0.41 |
| Angle LCA/C-tab or FSS, ° | 69.0 (65.0-72.7) | 68.8 (42.8-92.4) | 71.4 (61.7-78.7) | 0.44 | 70.4 (42.8-92.4) | 67.6 (55.9-72.7) | 0.38 |
| Angle LCA/THV NCC-LCC com, ° | 48.1 (42.2-55.2) | 48.8 (30.5-73.3) | 47.5 (30.4-58.7) | 0.41 | 47.0 (30.4-73.3) | 49.6 (32.4-57.7) | 0.56 |
| Coronary overlap – RCA   1. Optimal 2. Moderate 3. Severe | 27 (93)  2 (7)  0 (0) | 17 (89.5)  2 (10.5)  0 (0) | 10 (100)  0 (0)  0 (0) | 0.53  0.53  NA | 19 (90.5)  2 (9.5)  0 (0) | 8 (100)  0 (0)  0 (0) | 1.0  1.0  NA |
| Coronary overlap – LCA   1. Optimal 2. Moderate 3. Severe | 23 (79.3)  6 (20.7)  0 (0) | 17 (89.5)  2 (10.5)  0 (0) | 6 (60)  4 (40)  0 (0) | 0.14  0.14  NA | 16 (76.2)  5 (23.8)  0 (0) | 7 (87.5)  1 (12.5)  0 (0) | 0.65  0.65  NA |
| Optimal coronary overlap – RCA/LCA | 22 (75.9) | 16 (84.2) | 6 (60) | 0.19 | 15 (71.4) | 7 (87.5) | 0.64 |

Values are n (%), or median (range)

THV, transcatheter heart valve; RCA, right coronary artery; LCA, left coronary artery; FSS, free stent strut; NCC, non-coronary cusp; RCC, right coronary cusp; LCC, left coronary cusp; com, commissure.

**Supplemental Table 7.** THV alignment – virtual scenario #3 (see methods).

|  | **Overall**  n=29 | **CoreValve Evolut**  n=19 | **Acurate Neo2**  n=10 | **P value** | **Bicommissural type**  n=21 | **Tricommissural type**  n=8 | **P value** |
| --- | --- | --- | --- | --- | --- | --- | --- |
| Angle RCA/C-tab or FSS, ° | 74.8 (35.6-119.5) | 72.8 (35.6-119.5) | 78.5 (62.1-107.3) | 0.23 | 75.5 (35.6-119.5) | 74.7 (62.1-80.3) | 0.41 |
| Angle RCA/THV NCC-RCC com, ° | 45.2 (0.54-84.4) | 47.2 (0.54-84.4) | 41.5 (12.7-57.9) | 0.23 | 44.5 (0.54-84.4) | 45.3 (39.7-57.9) | 0.41 |
| Angle LCA/C-tab or FSS, ° | 59.3 (42.7-103.4) | 60.1 (44.4-103.4) | 59.1 (42.7-82.0) | 0.82 | 58.9 (42.7-103.4) | 64.0 (44.4-69.7) | 0.88 |
| Angle LCA/THV NCC-LCC com, ° | 60.8 (16.6-77.3) | 59.9 (16.6-75.7) | 60.9 (38.0-77.3) | 0.82 | 61.1 (16.6-77.3) | 56.0 (50.3-75.7) | 0.88 |
| Coronary overlap – RCA   1. Optimal 2. Moderate 3. Severe | 18 (62)  9 (31)  2 (7) | 13 (68.4)  5 (26.4)  1 (5.3) | 5 (50)  4 (40)  1 (10) | 0.43  0.68  1.0 | 11 (52.4)  8 (38.1)  2 (9.5) | 7 (87.5)  1 (12.5)  0 (0) | 0.11  0.37  1.0 |
| Coronary overlap – LCA   1. Optimal 2. Moderate 3. Severe | 27 (93)  1 (3.5)  1 (3.5) | 18 (94.7)  0 (0)  1 (5.3) | 9 (90)  1 (10)  0 (0) | 1.0  0.35  1.0 | 19 (90.5)  1 (4.8)  1 (4.8) | 8 (100)  0 (0)  0 (0) | 1.0  1.0  1.0 |
| Optimal coronary overlap – RCA/LCA | 18 (62) | 13 (68.4) | 5 (50) | 0.43 | 11 (52.4) | 7 (87.5) | 0.11 |

Values are n (%), or median (range)

THV, transcatheter heart valve; RCA, right coronary artery; LCA, left coronary artery; FSS, free stent strut; NCC, non-coronary cusp; RCC, right coronary cusp; LCC, left coronary cusp; com, commissure.

**Supplemental Table 8.** THV alignment – virtual scenario #4 (see methods).

|  | **Overall**  n=29 | **CoreValve Evolut**  n=19 | **Acurate Neo2**  n=10 | **P value** | **Bicommissural type**  n=21 | **Tricommissural type**  n=8 | **P value** |
| --- | --- | --- | --- | --- | --- | --- | --- |
| Angle RCA/C-tab or FSS, ° | 74.8 (35.6-119.5) | 72.8 (35.6-119.5) | 78.5 (62.1-107.3) | 0.23 | 75.5 (35.6-119.5) | 74.7 (62.1-80.3) | 0.41 |
| Angle RCA/THV NCC-RCC com, ° | 47.0 (6.61-97.6) | 47.0 (6.61-97.6) | 48.1 (10.6-72.2) | 0.71 | 46.6 (6.61-97.6) | 48.3 (37.2-72.2) | 0.46 |
| Angle LCA/C-tab or FSS, ° | 59.3 (42.7-103.4) | 60.1 (44.4-103.4) | 59.1 (42.7-82.0) | 0.82 | 58.9 (42.7-103.4) | 64.0 (44.4-69.7) | 0.88 |
| Angle LCA/THV NCC-LCC com, ° | 56.5 (27.1-79.3) | 57.0 (29.4-73.5) | 54.7 (27.1-79.3) | 0.96 | 57.0 (27.1-79.3) | 56.5 (33.0-70.6) | 0.92 |
| Coronary overlap – RCA   1. Optimal 2. Moderate 3. Severe | 20 (69)  7 (24)  2 (7) | 13 (68.4)  5 (26.3)  1 (5.3) | 7 (70)  2 (20)  1 (10) | 1.0  1.0  1.0 | 13 (61.9)  6 (28.6)  2 (9.5) | 7 (87.5)  1 (12.5)  0 (0) | 0.37  0.64  1.0 |
| Coronary overlap – LCA   1. Optimal 2. Moderate 3. Severe | 26 (89.7)  3 (10.3)  0 (0) | 18 (94.7)  1 (5.3)  0 (0) | 8 (80)  2 (20)  0 (0) | 0.27  0.27  NA | 19 (90.5)  2 (9.5)  0 (0) | 7 (87.5)  1 (12.5)  0 (0) | 1.0  1.0  NA |
| Optimal coronary overlap – RCA/LCA | 17 (58.6) | 12 (63.2) | 5 (50) | 0.69 | 11 (52.4) | 6 (75) | 0.41 |

Values are n (%), or median (range)

THV, transcatheter heart valve; RCA, right coronary artery; LCA, left coronary artery; FSS, free stent strut; NCC, non-coronary cusp; RCC, right coronary cusp; LCC, left coronary cusp; com, commissure.
